# Promotion of Bone Formation by Red Yeast Rice in Experimental Animals: A Systematic Review and Meta-Analysis

**DOI:** 10.1155/2020/7231827

**Published:** 2020-08-08

**Authors:** Bin Wu, Jie-Feng Huang, Bang-Jian He, Chen-Wei Huang, Jian-Hua Lu

**Affiliations:** ^1^The First Clinical Medical College, Zhejiang Chinese Medical University, Hangzhou 310006, China; ^2^Department of Orthopaedics, The First Affiliated Hospital of Zhejiang Chinese Medical University, Hangzhou 310006, China

## Abstract

**Objective:**

To systematically evaluate the effects of red yeast rice (RYR) and its extract on bone formation in experimental animals and to provide reference data for clinical research on the treatment of osteoporosis.

**Methods:**

Chinese and English language databases, including Web of Science, PubMed, the Cochrane Library, Elsevier, Google Scholar, SpringerLink, Embase, China National Knowledge Infrastructure (CNKI), Weipu Chinese Sci-tech periodical full-text database (VIP), and Wanfang Data Knowledge Service Platform (Wanfang), were searched from their establishment to February 2020 using the following terms: “hongqu,” “red yeast rice,” “*Monascus purpureus*-fermented rice,” “bone mineral density,” “osteoblast,” “osteoporosis,” and “animal models.” After excluding nonrelevant articles, Review Manager 5.2 was used to evaluate article quality and to analyze the data. Outcome indicators included bone mineral density (BMD), osteoblast proliferation, and the expression of alkaline phosphatase (ALP).

**Results:**

A total of 11 randomized controlled trials were included in the meta-analysis, all of which were animal studies. Six studies included data on BMD, five on osteoblast proliferation, and six on the expression of ALP. The results of the meta-analysis showed that RYR can significantly improve BMD (standardized mean difference (SMD) = 3.12, 95% confidence interval (CI) 1.41 to 4.83, *P* = 0.0003), promote osteoblast proliferation (SMD = 1.64, 95% CI 1.04 to 2.23, *P* < 0.00001), and increase ALP expression in rats (SMD = 1.25, 95% CI 0.69 to 1.80, *P* < 0.00001).

**Conclusions:**

RYR can promote bone formation in experimental animals and may be useful for the treatment of osteoporosis.

## 1. Introduction

Osteoporosis, a disease characterized by bone loss and bone microstructural changes, affects all bones and is common in women after menopause and elderly men. In osteoporosis, bones show increased brittleness, which makes them prone to fracture, which adversely affects the patient's ability to perform activities of daily life. The worldwide incidence of osteoporotic fractures is high. In Europe in 2010, osteoporosis accounted for 2% of all cases of noncommunicable disease [1]. The mortality rate of patients is also high, particularly among those with hip fractures [2]. It is important to improve the quality of life and reduce the mortality rate of osteoporosis patients.

Presently, the only drug reported to promote bone formation for the treatment of osteoporosis is teriparatide. However, its safety and side effects are noteworthy. It increases the incidence of osteosarcoma in rats [3], and in a fracture-prevention trial, patients reported a range of side effects, such as nausea, headache, dizziness, arthralgia, and leg cramps [4, 5]. Moreover, it is expensive, with a cost for daily form of $3426.5 for a 28-day supply as of June 2019 [6]. Therefore, effective novel drugs that can promote bone formation are needed.

Red yeast rice (RYR), produced by fermentation of the mycelia of *Monascus purpureus* Went parasitizing *japonica* rice, has been used in traditional medicine in Asia for thousands of years. As a traditional Chinese medicine, its main effects include promoting blood circulation to remove blood stasis, strengthening the spleen, and promoting food digestion. In addition, it has shown efficacy for the treatment of injuries, lochia after delivery, indigestion, and other ailments. Statins are widely used to treat hyperlipidemia and cardiovascular diseases. Furthermore, rodent studies have shown that statins stimulate bone formation both *in vitro* and *in vivo* [7]. Seven substances isolated from RYR have been used in statins [8]. These studies suggest that RYR has potential as a bone-forming agent. In addition, a meta-analysis of studies using RYR to treat primary hyperlipidemia showed short-term beneficial lipid modifications [9]. The RYR components used in statins are active and may be able to promote bone formation.

There have been recent reports regarding the effects of RYR on bone formation [10, 12], but the outcome indicators differed among the studies. This prompted us to perform the present meta-analysis. To the best of our knowledge, this is the first meta-analysis of the effects of RYR on bone formation in experimental animals. We examined the outcome indicators of relevant literature studies and analyzed the data to provide reference information for future clinical studies.

## 2. Materials and Methods

### 2.1. Literature Retrieval

Chinese and English language databases, including Web of Science, PubMed, the Cochrane Library, Elsevier, Google Scholar, SpringerLink, Embase, China National Knowledge Infrastructure (CNKI), Weipu Chinese Sci-tech periodical full-text database (VIP), and Wanfang Data Knowledge Service Platform (Wanfang), were searched from their establishment to February 2020 using the following terms: “hongqu,” “red yeast rice,” “*Monascus purpureus*-fermented rice,” “bone mineral density,” “osteoblast,” “osteoporosis,” and “animal models.” The search terms used were adjusted to ensure efficient literature retrieval. For example, the terms used for PubMed were (1) (“hongqu” OR “red yeast rice” OR “*Monascus purpureus*-fermented rice”); (2) (“bone mineral density” OR “osteoblast” OR “osteoporosis”); (3) animal models; and (4) (1) AND (2) AND (3).

### 2.2. Inclusion Criteria

The inclusion criteria are as follows: (1) an RYR experimental group and a vehicle-treated, saline, or placebo control group and (2) clearly defined outcome indicator and outcome data that could be readily extracted.

### 2.3. Exclusion Criteria

The exclusion criteria are as follows: (1) review or conference report, (2) duplicate articles (only the most complete article was included in the analyses), and (3) data that could not be extracted from the article.

### 2.4. Data Extraction

After eliminating duplicate articles, two researchers independently screened and extracted the data; differences were resolved by discussion. The data extracted included first author, publication year, sample size, intervention measures, intervention time, and main outcome indicators.

### 2.5. Data Analyses

Review Manager 5.2 (Cochrane Collaboration, Oxford, UK) was used to analyze the data. As the measurement methods and units for the main outcome indicators differed among studies, standardized mean differences (SMDs) and 95% confidence intervals (CIs) were calculated for continuous outcomes. The *I*^2^ statistic was used as an index of the heterogeneity of the included studies. *I*^2^ values ≥ 50% suggest significant heterogeneity. In such cases, a random-effect model was used in the analyses. When there was no obvious heterogeneity, the fixed-effect model was used. In cases of heterogeneity, sensitivity analyses were performed by excluding articles one by one to find the source thereof. Funnel plots were used to determine whether there was publication bias; asymmetry in these plots indicates the presence of bias.

## 3. Results

### 3.1. Literature Screening

**Figure 1:**
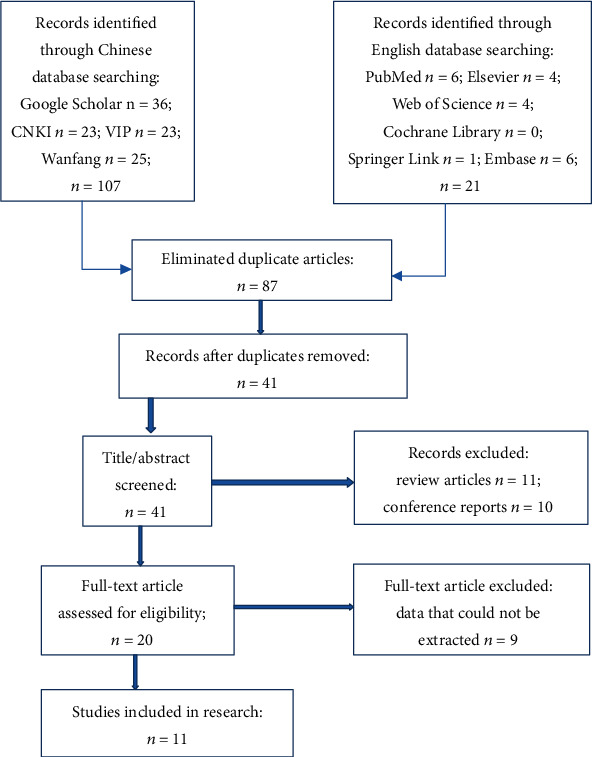
Literature screening process.

A total of 128 articles were retrieved. Eighty-seven duplicated articles were removed, and the titles and abstracts of the remaining articles were read. After excluding papers that did not meet the inclusion criteria, 11 randomized controlled studies were included in the analyses after careful screening. The article screening process is shown in Figure 1.

### 3.2. Basic Information of the Included Articles

The 11 articles included in the analyses all described animal experiments. The experimental groups received RYR or extracts thereof, while the control groups typically received saline or distilled water. The basic information of the included articles is shown in Table 1.

### 3.3. Evaluation of the Quality of Included Articles

**Figure 2:**
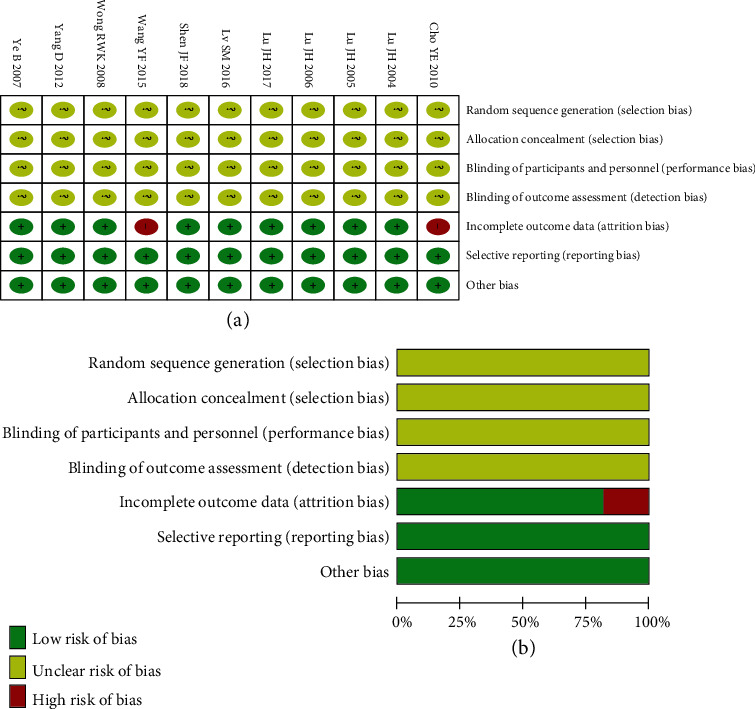
Evaluation of the quality of studies: the Cochrane Collaboration's tool for assessing the risk of bias in randomized trials.

The 11 articles included were all randomized controlled animal studies. No study described the randomization procedures; allocation concealment, blinding of the participants, and blinding of outcome assessment were not mentioned. Two articles with incomplete data were considered to have high risk of bias. The missing data on standard deviation (SD) were evenly distributed among the groups, and the reasons for its loss were similar. It was insufficient to influence the effect size estimation, so it was kept. The risk for bias for all articles is shown in Figure 2.

### 3.4. Meta-Analysis Results

#### 3.4.1. Bone Mineral Density

**Figure 3:**
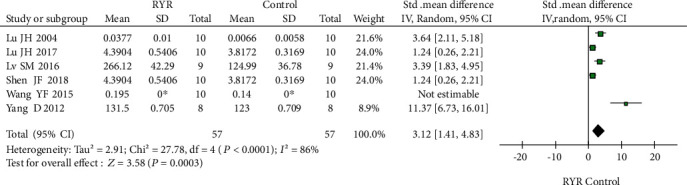
Forest plot comparing the BMD data of the RYR and control groups: the heterogeneity index *I*^2^ indicates the existence of heterogeneity. A random-effect model and sensitivity analyses were conducted, *P* < 0.001. ^∗^Standard deviation was shown in histogram, without exact data.

Bone mineral density (BMD) outcomes were reported in six articles (57 cases each in the RYR and control groups). The *I*^2^ value in the heterogeneity test was 86% (*P* < 0.0001). The random-effect model indicated that RYR significantly improved the BMD of rats compared to control animals (SMD = 3.12, 95% CI 1.41 to 4.83, *P* = 0.0003). *I*^2^ showed little change after exclusion of articles (one by one) in sensitivity analyses, and no particular source of heterogeneity was found. The results are shown in detail in Figure 3.

#### 3.4.2. Osteoblast Proliferation

**Figure 4:**
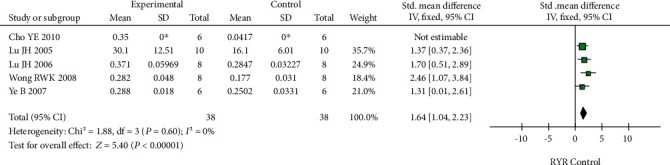
Forest plot comparing the osteoblast proliferation data of the RYR and control groups: the heterogeneity index *I*^2^ indicated no obvious heterogeneity. A fixed-effect model suggested *P* < 0.00001.

**Figure 5:**
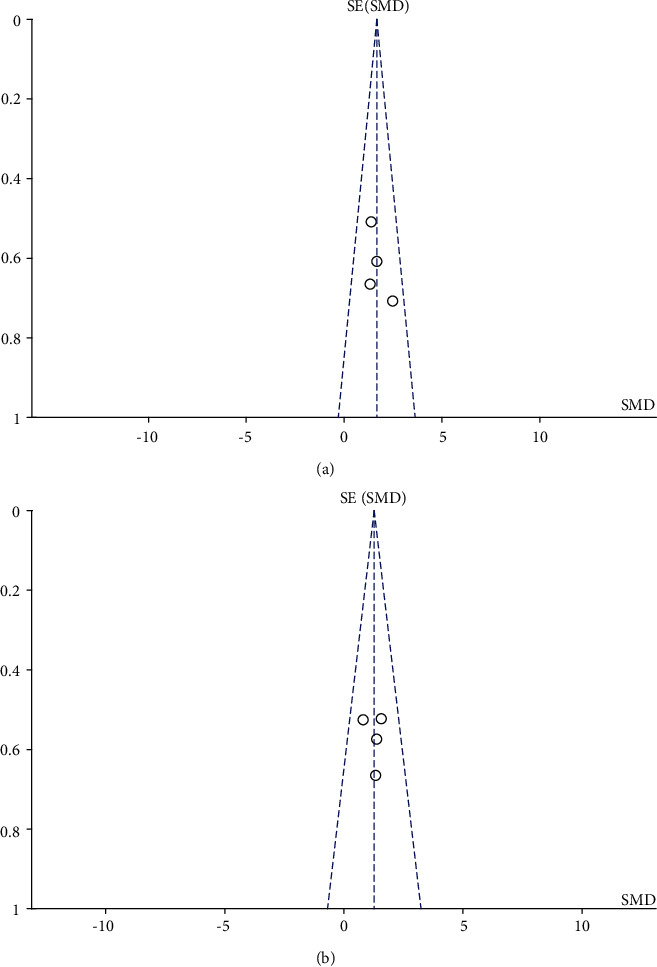
Funnel plots of (a) osteoblast proliferation and (b) ALP expression: both were symmetric, indicating no obvious publication bias.

Osteoblast proliferation was assessed in five studies (38 cases each in the RYR and control groups). The *I*^2^ value in the heterogeneity test was 0% (*P* = 0.60), indicating no significant heterogeneity. Thus, a fixed-effect model was used for the analyses. The results suggested that RYR significantly promoted osteoblast proliferation compared to the controls (SMD = 1.64, 95% CI 1.04 to 2.23, *P* < 0.00001). The results are shown in detail in Figure 4. The funnel plot was symmetrical, indicating no obvious publication bias (Figure 5).

#### 3.4.3. Expression of Alkaline Phosphatase

**Figure 6:**
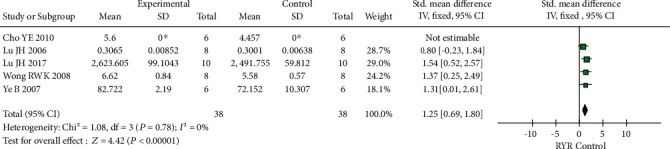
Forest plot comparing the ALP expression data of the RYR and control groups: the heterogeneity index *I*^2^ indicated the existence of heterogeneity. A random-effect model and sensitivity analyses were used to eliminate the sources of heterogeneity. Then, a fixed-effect model suggested *P* < 0.00001.

Six trials assessed alkaline phosphatase (ALP) expression (38 cases each in the RYR and control groups). The *I*^2^ value in the heterogeneity test was 74% (*P* = 0.004). The random-effect model indicated no significant difference between the RYR and control groups in ALP expression (*P* = 0.12). Sensitivity analyses indicated that one article was the source of the heterogeneity. After removing this article, the *I*^2^ value was 0% (*P* = 0.78), and the fixed-effect model showed indicated a significant difference in ALP expression between the groups (SMD = 1.25, 95% CI 0.69 to 1.80, *P* < 0.00001). The results obtained after excluding the article responsible for the heterogeneity are shown in Figure 6. The funnel plot was relatively symmetrical, indicating no obvious publication bias (Figure 5).

## 4. Discussion

The interaction between osteoblasts and osteoclasts is important for bone formation and bone absorption. Under the regulation of various cytokines and through complex molecular mechanisms, these two cell types jointly maintain the balance of bone metabolism. When osteoblasts are active, bone formation predominates over bone resorption. The process of bone formation can be divided into several stages, i.e., osteoblast proliferation, extracellular matrix maturation, matrix mineralization, and osteogenesis. These processes are regulated by various factors, and osteoblasts serve as important functional cells. Stem cells, as immature and incompletely differentiated cells, can differentiate into various cell types under the control of regulatory factors. Osteoblasts are also differentiated from mesenchymal stem cells [22]. Two signaling pathways play important roles in the differentiation of mesenchymal stem cells into osteoblasts, i.e., the Wnt/*β*-catenin signaling pathway and the BMP-2/Smad signaling pathway. Activation of the Wnt signaling pathway can increase the expression of BMP-2 [23]. Some groups have also demonstrated that the Wnt signaling pathway can be regulated by activation of the BMP signaling pathway [24]. Runx2 is expressed in both pathways and acts on key regulators and plays an important role in differentiation.

ALP is a glycoprotein widely distributed in various tissues in the body. ALP levels are higher in newborn and growing children, and ALP is highly expressed during osteoblast differentiation [25]. Increased Runx2-regulated ALP activity in the Wnt signaling pathway is an important marker for differentiation of mesenchymal stem cells into osteoblasts [26]. High ALP expression levels reflect the proliferation of osteoblasts, which have many functions including the synthesis and secretion of bone matrix, and mineralization of bone matrix to form new bone. Osteoblasts secrete bone matrix (mainly type I collagen) to control the flow of calcium, phosphorus, and magnesium and in turn regulate mineralization. ALP can promote mineralization, has been shown to release inorganic phosphorus during hydrolysis of organophosphorus, and is considered to play an important role in the removal of inorganic pyrophosphate, which is an effective inhibitor of mineralization [27]. Matrix mineralization is a marker of osteoblast differentiation and maturation. BMD is an indicator of bone strength; osteoporosis occurs when BMD is below normal, increasing the risk of fracture. When the process of extracellular matrix mineralization is complete and new bone has formed, BMD also increases.

Osteoporosis has a high incidence and mortality rate, so its prevention and treatment are important. The guidelines for diagnosing and treating primary osteoporosis specify basic measures, drug interventions, and rehabilitation as the main strategies. Osteoporosis is treated with drugs that inhibit bone resorption and promote bone formation, among other actions.

In this analysis, we found that RYR and its extract promote the proliferation of osteoblasts, which is important in bone formation. It may activate the signaling pathway by increasing the BMP content of mesenchymal stem cells, causing them to differentiate into osteoblasts. We found that RYR could also increase the expression of ALP, which is related to the proliferation of osteoblasts. RYR-induced improvement in BMD may be related to the promotion of bone matrix mineralization by osteoblasts. Compared to most bone absorption inhibitors used to treat osteoporosis, RYR has the advantages of promoting the proliferation of osteoblasts, thereby promoting bone formation, and increasing the mass and strength of bone tissue. Therefore, it should become a new drug for promoting bone formation.

Parathyroid analogs are representative promoters of bone formation and have been shown to bind to Runx2 and upregulate its transcription [28]. Intermittent use of small doses of these drugs can increase the activity of osteoblasts and thus promote bone formation. Teriparatide [rhPTH(1–34)] is the only drug currently available that promotes bone formation, through the active fragment of human endogenous parathyroid hormone. This drug directly stimulates osteoblasts to produce new bone tissue, thereby increasing bone mass and strength [29]. It can significantly reduce the risk of vertebral and nonvertebral fractures in postmenopausal women [30, 31]. In addition, it is often used to treat postmenopausal women with osteoporosis. Compared to teriparatide, RYR, as a natural compound, has few side effects and costs only $1.27 per kilogram in Chinese market, which makes it suitable for the development of new drugs that may contribute to the treatment of osteoporosis in the future.

## 5. Conclusion

The results of this meta-analysis showed that RYR and its extract can promote BMD, osteoblast proliferation, and ALP expression in rats and thus, by extension, bone formation. All of the articles included in this meta-analysis were randomized controlled animal studies, with high evidence and reliability levels. The results indicate that RYR promotes bone formation and therefore has potential for the treatment of osteoporosis. However, the articles included were in different languages and the sample size was small; thus, further clinical studies are needed to confirm the efficacy of RYR.

## Figures and Tables

**Table 1:** Basic information of the articles.

Included studies	Group size		Intervention measures		Intervention time	Outcome indicators
	RYR	Control	RYR	Control		
Wang et al. 2015 [11]	10	10	6.24 g/kg RYR extract	10 mL/kg saline	20 w	(1)
Wong and Rabie 2008 [12]	8	8	RYR medium	Blank control medium	48 h	(2)(3)
Cho et al. 2010 [13]	6	6	RYR medium	Blank control medium	5 d	(2)(3)
Lu et al. 2006 [14]	8	8	10 mL/kg RYR extract	10 mL/kg saline	10 d	(2)(3)
Lu et al. 2017 [15]	10	10	10 mL/kg RYR extract	10 mL/kg distilled water	12 w	(1)(3)
Lu et al. 2004 [16]	10	10	10 mL/kg RYR extract	10 mL/kg saline	12 w	(1)
Lu et al. 2005 [17]	10	10	10 mL/kg RYR extract	10 mL/kg saline	12 w	(2)
Shen et al. 2018 [18]	10	10	10 mL/kg RYR extract	10 mL/kg 0.9% sodium chloride solution	12 w	(1)
Lv et al. 2016 [19]	9	9	0.5 RYR capsule/kg	5 mL/kg distilled water	60 d	(1)
Yang and Zhang 2012 [20]	8	8	RYR extract	Saline	12 w	(1)(3)
Ye and Yang 2007 [21]	6	6	RYR medium	Blank control medium	72 h	(2)(3)

(1): bone mineral density (BMD); (2): osteoblast proliferation; (3): alkaline phosphatase (ALP) expression.
